# Do Chinese Children Get Enough Micronutrients?

**DOI:** 10.3390/nu9040397

**Published:** 2017-04-18

**Authors:** Huijun Wang, Dantong Wang, Yifei Ouyang, Feifei Huang, Gangqiang Ding, Bing Zhang

**Affiliations:** 1 National Institute for Nutrition and Health, Chinese Center for Disease Control and Prevention, Beijing 100050, China; wanghj128@gmail.com (H.W.); hollyfaye@126.com (Y.O.); fayekobe@163.com (F.H.); dinggq@chinacdc.cn (G.D.); 2 Nestlé Research Center, Lausanne CH-1000, Switzerland; Dantong.Wang@rdls.nestle.com

**Keywords:** micronutrients, inadequacies, usual daily intake, China

## Abstract

The aim of this study was to examine usual daily micronutrient intake of Chinese children based on data from the 2011 China Health and Nutrition Survey. We analyzed data from 4 to 17-year-old participants, who provided dietary data on three consecutive days combined with the household weighing method in 2011. Usual daily intake of each nutrient was estimated using a mixed effects model based on the China Food Composition published in 2009. The means, medians and percentages below Estimated Average Requirements (EAR) were reported for selected micronutrients, including calcium, sodium, potassium, iron, zinc, selenium, vitamin A, thiamine, riboflavin and vitamin C. For sodium and potassium, the means and the distribution of intakes were compared to the Adequate Intake (AI) level. The average usual daily intakes of all micronutrients increase with age, and the intakes of boys were found to be higher than girls in the same age group. The average calcium intake increased from 272 mg/day in 4–6 years to 391 mg/day in 14–17 years, but the percentage of inadequate calcium intake remained very high (>96%). The prevalence of inadequacy of calcium was the highest among the mineral nutrients reported in this study. As the requirements of micronutrients increased with age, the percentage of subjects with inadequate intake increased in the 11–17 years age groups. Among 14–17 years group, the percentages of study participants with dietary intakes of calcium, iron, zinc, selenium, vitamin A, thiamine, riboflavin and vitamin C below the EAR were 96.8%, 18.8%, 37.6%, 72.8%, 36.8%, 91.8%. 85.9% and 75.5%, respectively. Among 11–13 years group, the percentages of study participants with dietary intakes of iron, zinc and vitamin A below the EAR were 23.5%, 41.5%, and 41.6%, respectively. Thus, micronutrient deficiency is a problem in Chinese children. Nutrition education and intervention programs are needed to address these nutritional gaps.

## 1. Introduction

Deficiencies of micronutrients are prevalent among children in developing as well as some developed countries [1,2,3,4]. Low micronutrient intake may link to long-term health risks [5,6,7]. Recent European studies have suggested that substantial numbers of European children might be at risk due to inadequate intake of micronutrients [4,8]. China’s dietary changes are enormous [9,10], and the shifts in foods consumed and eating behaviors will continue to accelerate [11]. Recent shifts toward increased snacking [12], away-from-home eating [13], and modern packaged processed foods [14] present dietary challenges in the younger Chinese generations. Low consumption of vegetables, fruits [15], and dairy products [16] affect the nutrition status of children. It is common to see problems of underweight, stunting and micronutrient deficiencies in parallel with an increasing prevalence of overweight and obesity in Chinese children and adolescents. The public health and nutrition policy in China has been focused on providing an adequate dietary supply to decrease the prevalence of malnutrition in Chinese children [17]. However, it is still unclear how many Chinese children meet the recommended intakes of micronutrients at the national level.

The most recent Chinese Dietary Reference Intakes (DRIs) were published in 2013 [18]. The DRIs allow for a comparison between daily intakes and recommendations to assess the adequacy level of macro- and micronutrient intake. The China Health and Nutrition Survey (CHNS) is a longitudinal study starting from 1991; food intake data were collected using 24-h recalls on three consecutive days, combined with the household weighing method [19]. Based on CHNS dietary intake data collected in 2011, we examined micronutrient intakes from childhood to adolescence to answer the question: Do Chinese children get enough micronutrients?

## 2. Materials and Methods

### 2.1. CHNS and Study Subjects

This study was based on data from the CHNS, an ongoing longitudinal survey that has been conducted in 1991, 1993, 1997, 2000, 2004, 2006, 2009 and 2011. It was designed to provide a representative sample of households in China by geographical location, level of economic development and use of public resources. The survey focused on examining household- and individual-level socio-demographic factors, diet, physical activity, health status and behavior changes. Detailed information can be found on the project website. We analyzed data from 4- to 17-year-old participants, in total 1905 children who had diet data on three consecutive days in 2011, and stratified the data by gender and age.

### 2.2. CHNS Dietary Measurement Methods

Individual dietary data for three consecutive days (two weekdays and one weekend day) were recorded for all household members 2 years and older. This was achieved by asking each individual, except children younger than 12 years, to report all food consumed at home and away from home in the past 24 h. For children younger than 12 years, the mother or a caregiver who handled food preparation and feeding in the household was asked to recall the children’s food consumption. Using food models and picture aids, trained field interviewers recorded the type of food, amount, and type of meal and place of consumption of all food items during the past 24 h. Household edible oils, sugar and salt consumption were determined on a daily basis by calculating the changes in home food inventory by weighing [19]. Dietary intake data were derived from 24-h recalls on three consecutive days, combined with the household weighing method. Food recalls were coded and analyzed to calculate nutrient intakes using the Chinese Food Composition Tables published in 2009 [20]. We reported the results on the intake of key micronutrients, including calcium, sodium, potassium, iron, zinc, selenium, vitamin A, thiamine, riboflavin and vitamin C.

### 2.3. Micronutrient Analysis

The estimated average requirement (EAR) cut-point method was used to estimate the proportion of individuals in the group at the risk of having inadequate micronutrient intakes [21,22,23]. For sodium and potassium, where the EARs for Chinese children have not yet been established due to insufficient information, the means and distribution of intakes were compared to the Adequate Intake (AI) level. If a group has a mean or median intake at or above the AI, there is a low prevalence of low intake levels. If the mean or median is less than the AI, no inferences about the prevalence of low intake levels were made [21,23]. Appendix A Table A1 provides the EAR or AI for each nutrient by life stage group [18].

### 2.4. Usual Daily Intake Estimation

Usual daily intake is defined as the long-run average daily intake of a dietary component by an individual. Usual daily intake of each nutrient was estimated by the mixed effects model and quantile estimation procedure, developed at the National Cancer Institute (NCI). It is one of the statistical procedures for estimating the usual intake distribution from repeated 24-h recalls [24,25]. A separate model was created for each nutrient.

### 2.5. Statistical Analysis

In our analysis, micronutrient intake was assessed in four age groups of girls and boys (aged 4 to 6 years, 7 to 10 years, 11 to 13 years and 14 to 18 years). Continuous variables were expressed as the means, median, 25th percentile and 75th percentile (P25, P75), whereas categorical variables were expressed as percentages (%). Statistical analysis was done with SAS 9.2 (SAS Institute Inc., Cary, NC, USA).

## 3. Results

### 3.1. Characteristics of the Study Subjects

As shown in Table 1, in total 1905 children aged 4 to 17 years were included in the study. The average age was 8.5 and 51.8% of the subjects were boys.

### 3.2. Characteristics of the Study Subjects

Table 2 summarizes the mean and selected percentiles of usual dietary intakes, and the percentages of children with inadequate nutrient intakes of calcium, iron, zinc and selenium. Average usual dietary intakes of these minerals were found to increase with age and the intakes of boys were slightly higher than girls in the same age group. The calcium intake increased from 272 mg/day in the 4–6 years group to 391 mg/day in the 14–17 years group, but the percentage of inadequate calcium intake remained very high (>96%) in all groups. As iron intake increased with age among boys, the percentage of inadequate iron intake decreased. Although iron intake also increased with age in girls, due to the increasing dietary iron requirements, the percentage of inadequate iron intake also increased with age in girls. The zinc intake increased from 6.3 mg/day in the 4–6 years group to 10.0 mg/day in the 14–17 years group, and the percentage of inadequate zinc intake was higher in girls than in boys. The intake of selenium increased with age from 27.0 mg/day (4–6 years) to 45.6 mg/day (14–17 years). However since the requirements for dietary selenium also increased with age, the prevalence of inadequate selenium intake was also higher among older children (at about 50% in the 4–6 years group and 72.8% in the 14–17 years group).

Usual intakes of both sodium and potassium increased with age, and the intakes of boys were a little higher than girls in the same age group (Table 3). The mean and median intakes of sodium were much higher than the AI for each respective age group. For example, the median of sodium in the 14–17 years group, 4330 mg/day, was much higher than AI of 1600 mg/day. On the contrary, the mean and median intakes of potassium were less than the AI for each respective age group. The gaps between the recommendation and the mean and median intakes grew wider as age increased.

The average and selected percentiles of usual dietary intakes of vitamin A, thiamine (vitamin B1), riboflavin (vitamin B2) and vitamin C are shown in Table 4. Average intakes of these vitamins were found to increase with age, and the intakes in boys were higher than in girls in the same age group. As the requirements of vitamin A, thiamine, riboflavin and vitamin C increase as children get older, the percentage of subjects with inadequate intake also increased with age. In the 14–17 years group, the percentage of study participants with dietary intakes of vitamin A, thiamine, riboflavin and vitamin C below the EAR were 36.8%, 91.8%. 85.9% and 75.5%, respectively. In the 11–13 years group, the percentages of study participants with dietary intakes of vitamin A, thiamine, riboflavin and vitamin C below the EAR were 41.6%, 89.1%, 83.7% and 69.0%, respectively.

## 4. Discussion

This is the first study that used usual nutrient intake estimations and applied the EAR cut-point method to assess the adequacy of micronutrient intakes among 4 to 17-year-old children and adolescents in China [23,24]. Previously, researchers reported low intakes of calcium [26], iron [27] and vitamin C [28] in Chinese children based on average intake values, but no prevalence of inadequate intake was calculated. In one study, the usual nutrient intakes and prevalence of inadequate intakes were estimated in adults based on the data from China National Nutrition and Health Survey in 2002. Results highlighted the inadequate intakes of some micronutrients, such as calcium, zinc, selenium, thiamine and riboflavin [29]. The results of this study are consistent with previous reports [26,27,28].

Although the dietary patterns in China have changed in the past 20 years [30], Chinese children do not get enough micronutrients. The present study showed that, compared to the current reference values, there was a substantial percentage of Chinese children who had low intakes for calcium, zinc, selenium, vitamin A, thiamine, riboflavin and vitamin C. The prevalence of inadequate nutrient intakes ranged from 9.7% in the 4–6 years group for iron to 98.6% in the 11–13 years group for calcium. For the majority of nutrients analyzed in this study, nutrient intake increased with age, which is to be expected, since older children consume more food and have higher energy intakes [31]. However, the prevalence of inadequacy also increased because older children have increased requirements growing up.

The prevalence of inadequate calcium intake was the highest among the mineral nutrients analyzed in this study. The main reason for this is likely the low consumption of dairy products, which is the most important food source of dietary calcium in Chinese children [16]. Although the percentage of inadequate iron was the lowest among the mineral nutrients, we should pay attention to girls’ intake. For girls over 10 years old, the iron EAR cut-points were higher than boys while the intake level was lower, thus the prevalence of inadequacy of iron showed a significant gender difference. Among 11–13 year olds, the prevalence of inadequate iron intake in girls was 31.2 points higher than in boys. There were also high proportions of children and adolescents who had intakes below the EAR for zinc and selenium, ranging from 24.2% to 41.5% for zinc and from 50.8% to 72.8% for selenium, respectively. Compared with the children in European countries, the prevalence of calcium, iron, selenium and zinc inadequacies were much higher in Chinese children. For example, in children aged 4–10 years in European countries, calcium intakes ranged from 563 mg/day in Polish girls to 1106 mg/day in Danish boys, with only 32% of the girls and 28% of the boys having calcium intakes below the EAR [8,32]. Compared with the children in the U.S., based on America, National Health and Nutrition Examination Survey (NHANES) 2007–2010, calcium intakes were much higher than Chinese children; calcium intakes were 975 mg/day and 1047 mg/day in U.S. children aged 4–8 years and 9–18 years, respectively. Calcium inadequacies were much lower than Chinese children; in U.S. children, only 5.8% of the boys aged 4–8 years showed calcium intakes below the EAR, but the percentage was also very high in some age groups, for example, the percent was 81.9% of the girls aged 9–18 years in U.S. [33].

The mean and median intakes of potassium were below the AI, but no clear conclusions can be drawn about the prevalence of low intakes [21]. We found that the gap between potassium mean intakes and AI increased as age increased. In European countries, potassium intakes among children were much higher than in Chinese children. In 4–10 year olds in European countries, the mean intake ranged from 2077 mg/day (Dutch girls aged 4–6 years) to 3044 mg/day (Dutch boys aged 7–10 years); the mean intake among 11–17 year olds ranged from 2148 mg/day (UK girls) to 3899 mg/day (German boys) [32]. In the U.S., although less than 2% of Americans in any age-gender group consume at or above the AI for potassium, potassium intakes among children were much higher than in Chinese children. Based on NHANES 2007–2010, the means of potassium intake were 2049 mg/day in those aged 4–8 years and 2261 mg/day in those aged 9–18 years [33]. Sodium intakes were much higher than the AI for each respective age group. There is a low prevalence of low intake levels, but we should also pay attention to the health effects of high sodium intakes.

The intake levels of thiamine and riboflavin found in children and adolescents in this study were very similar to the reported intake distribution of adults in China, based on the China National Nutrition and Health Survey in 2002. The prevalence of inadequacies of both vitamins was over 80% [29]. Cereal products are one of the most important sources of thiamine, but the consumption of cereal products has decreased in Chinese children over the past twenty years [30]. Perhaps this is one of the main reasons for the increasing prevalence of inadequate thiamine intake. In children aged 4–17 years European countries, inadequate intakes of thiamine and riboflavin are low. The highest proportion of intakes below the EAR was for thiamine (8% in girls age 4–10 years from Poland), and for riboflavin (26% observed in girls aged 11–17 years in UK) [32]. In China, high percentages of children and adolescents had inadequate intakes for vitamin C and vitamin A, ranging from 53.9% to 75.5% (vitamin C) and from 25.3% to 36.8% (vitamin A), and the proportions increased with age. The percentages of inadequate vitamin C intake were higher in Chinese children than among European children (0.2% to 8.0% in European children). The proportion of intakes below the EAR for vitamin A varied among the European countries, ranging from 2% for Germany to 30% for UK children aged 11–17 years [32]. In the U.S., vitamin A and riboflavin deficiencies were not widespread concerns [33].

Greater dietary diversity has been associated with micronutrient status in many studies [34,35]. Improving diet variety and quality to meet the nutrient intake recommendations may be an effective approach to reduce health risks in Chinese children. Geographic location and socioeconomic status vary a lot in China, and this could have an important impact on dietary habits, food quality, and micronutrient intake. Further studies are needed to discover the differences and provide evidence for developing community-specific strategies to improve micronutrient intake.

## 5. Conclusions

Based on the usual daily micronutrient intake of Chinese children which came from the 2011 China Health and Nutrition Survey, Chinese children do not get enough micronutrients. The present study showed that, compared to the current reference values, there was a substantial percentage of Chinese children who had low intakes for calcium, zinc, selenium, vitamin A, thiamine, riboflavin and vitamin C. micronutrient deficiency is a problem in Chinese children. Nutrition education and intervention programs are needed to address these nutritional gaps.

## 6. Limitations

Dietary supplement consumption data were not collected in CHNS 2011, but the effect is likely to be small. To our knowledge, there are few studies focused on dietary supplement use in the China. One study indicated that the intake rate of vitamin and mineral dietary supplements among 3 to 12-year-old children in seven cities and two counties ranged widely, from 27.46% for calcium to 4.87% for iron [36]. Rates of supplement use were lower compared to other countries, for example in Korea, where dietary supplement use is 49.5%–54.2% among 1 to 6-year-olds [37] and 28.5% in 9 to 18-year-olds [38]. One-third of 4 to 18-year-old children in the United States were reported to use dietary supplements from 2003 to 2006 [39]. So, it is possible that this study slightly underestimated vitamin and mineral intakes in Chinese children. Another limitation is the risk of under-reporting. A European study assessed the impacts of under-reporting micronutrient intakes. After excluding under-reporters, the mean intake increased slightly in some age groups, but the inadequate intake level did not change substantially [32]. Therefore, we estimate the impact of potential under-reporting could be limited.

## Figures and Tables

**Table 1:** Characteristics of the study subjects. SD, standard deviation.

Items	Gender		Total
	Boys	Girls	
Age (years, Mean ± SD)	8.0 ± 4.9	8.5 ± 4.8	8.2 ± 4.9
4–6	53.1%	46.9%	461
7–10	50.2%	49.8%	623
11–13	53.8%	46.2%	405
14–17	50.7%	49.3%	416
Urban	51.7	48.3	755
Rural	50.3	49.7	1150
Total	51.8%	48.2%	1905

**Table 2:** Usual daily intake and proportion below the estimated average requirements (EAR) for calcium, iron, zinc and selenium among Chinese children.

Items	Nutrients											
	Calcium (mg/Day)			Iron (mg/Day)			Zinc (mg/Day)			Selenium (mg/Day)		
	Mean	Median (P25, P75)	Below EAR (%)	Mean	Median (P25, P75)	Below EAR (%)	Mean	Median (P25, P75)	Below EAR (%)	Mean	Median (P25, P75)	Below EAR (%)
Boys												
4–6	273	237 (164, 338)	97.1	12.4	11.7 (9.1, 14.9)	7.8	6.7	6.3 (5.0, 8.0)	18.9	27.6	25.7 (18.7, 34.3)	47.3
7–10	314	273 (190, 389)	97.9	15.6	14.6 (11.5, 18.6)	14.6	8.0	7.6 (6.0, 9.6)	23.9	34.2	32.4 (24.1, 42.7)	57.5
11–13	378	328 (229, 469)	98.1	19.0	17.8 (14.0, 22.6)	9.2	9.5	9.0 (7.1, 11.3)	39.3	40.6	37.9 (28.5, 49.7)	66.1
14–17	428	374 (261, 532)	96.2	21.5	20.3 (15.9, 25.7)	7.2	11.2	10.7 (8.5, 13.4)	39.0	46.8	44.1 (33.3, 57.2)	62.9
Girls												
4–6	269	235 (160, 339)	97.5	11.4	10.7 (8.4, 13.6)	11.4	5.9	5.6 (4.4, 7.1)	29.7	26.1	23.8 (16.9, 32.8)	53.9
7–10	312	274 (188, 390)	97.9	14.3	13.5 (10.6, 17.0)	20.0	7.4	7.1 (5.6, 8.9)	30.4	29.7	27.4 (19.7, 37.1)	70.6
11–13	345	301 (208, 430)	98.9	16.2	15.2 (12.0, 19.3)	40.4	8.4	8.0 (6.3, 10.0)	44.1	34.8	32.1 (23.2, 43.2)	78.2
14–17	360	317 (218, 450)	97.6	17.7	16.7 (13.1, 21.0)	30.8	8.8	8.5 (6.7, 10.5)	36.1	36.8	34.2 (24.9, 45.7)	81.4
Total												
4–6	272	236 (163, 338)	97.1	12.0	11.2 (8.8, 14.3)	9.7	6.3	6.0 (4.6, 7.6)	24.2	27.0	24.8 (17.8, 33.7)	50.8
7–10	313	273 (189, 390)	97.9	14.9	14.0 (11.0, 17.8)	17.7	7.7	7.3 (5.7, 9.2)	27.8	32.2	29.8 (21.6, 39.9)	64.3
11–13	364	317 (219, 454)	98.6	17.4	16.6 (13.0, 21.2)	23.5	9.0	8.6 (6.7, 10.8)	41.5	38.1	35.3 (25.8, 47.1)	71.4
14–17	391	342 (237, 488)	96.8	19.5	18.3 (14.3, 23.3)	18.8	10.0	9.5 (7.5, 11.9)	37.6	45.6	38.7 (28.6, 51.3)	72.8

**Table 3:** Mean and selected percentiles of usual daily sodium and potassium intakes among Chinese children.

Items	Nutrients							
	Sodium (mg/Day)				Potassium (mg/Day)			
	Mean	P25	Median	P75	Mean	P25	Median	P75
Boys								
4–6	3267	1572	2782	4400	1066	748	987	1289
7–10	39,786	2082	3463	5302	1318	929	1217	1586
11–13	4586	2502	4037	6055	1567	1110	1451	2187
14–17	5150	2923	4607	6769	1776	1264	1652	2146
Girls								
4–6	3022	1592	2619	4016	998	702	927	1214
7–10	3632	2012	3191	4743	1208	858	1125	1460
11–13	3799	2016	3329	4948	1307	989	1287	1674
14–17	4598	2690	4124	5961	1387	1021	1287	1732
Total								
4–6	3148	1591	2691	4189	1035	725	957	1254
7–10	3787	2032	3315	4995	1259	887	1169	1523
11–13	4228	2323	3721	5563	1487	1047	1378	1802
14–17	4849	2781	4330	6344	1593	1127	1480	1930

**Table 4:** Usual daily intake and proportion below the EAR for vitamin A, thiamine, riboflavin and vitamin C among Chinese children.

Items	Nutrients											
	Vitamin A (µg/Day) *			Thiamine (mg/Day)			Riboflavin (mg/Day)			Vitamin C (mg/Day)		
	Mean	Median (P25, P75)	Below EAR (%)	Mean	Median (P25, P75)	Below EAR (%)	Mean	Median (P25, P75)	Below EAR (%)	Mean	Median (P25, P75)	Below EAR (%)
Boys												
4–6	555	443 (263, 714)	24.6	0.6	0.5 (0.4, 0.7)	61.6	0.6	0.5 (0.4, 0.7)	61.9	45.2	39.7 (25.2, 58.8)	50.5
7–10	645	520 (315, 831)	30.8	0.6	0.6 (0.5, 0.8)	76.6	0.7	0.6 (0.4, 0.8)	74.2	55.1	49.0 (32.2, 71.1)	57.9
11–13	734	592 (362, 941)	38.6	0.8	0.7 (0.6, 0.9)	87.9	0.8	0.7 (0.5, 0.9)	85.6	67.9	61.0 (41.2, 86.8)	65.0
14–17	872	715 (441, 1121)	39.3	0.9	0.8 (0.7, 1.1)	89.7	0.9	0.8 (0.6, 1.1)	86.8	70.7	64.0 (43.5, 90.4)	70.8
Girls												
4–6	487	407 (250, 635)	26.7	0.5	0.5 (0.3, 0.6)	75.9	0.5	0.5 (0.3, 0.6)	70.3	40.6	35.6 (22.6, 53.2)	57.4
7–10	604	511 (321, 778)	30.3	0.6	0.6 (0.4, 0.7)	82.7	0.6	0.6 (0.4, 0.7)	79.9	54.8	49.1 (32.6, 70.4)	58.2
11–13	582	488 (306, 749)	45.1	0.7	0.6 (0.5, 0.8)	90.6	0.7	0.6 (0.5, 0.8)	81.5	59.5	53.3 (35.7, 76.2)	73.9
14–17	688	588 (373, 887)	34.3	0.7	0.7 (0.5, 0.8)	94.0	0.7	0.7 (0.5, 0.9)	85.0	61.3	55.5 (37.3, 78.8)	79.6
Total												
4–6	522	426 (259, 674)	25.3	0.5	0.5 (0.4, 0.6)	68.3	0.5	0.5 (0.4, 0.7)	66.0	43.0	37.6 (24.0, 55.9)	53.9
7–10	624	518 (318, 804)	30.6	0.6	0.6 (0.4, 0.8)	79.4	0.6	0.6 (0.4, 0.8)	77.1	54.9	49.1 (32.3, 70.7)	58.1
11–13	661	547 (337, 853)	41.6	0.7	0.7 (0.5, 0.9)	89.1	0.7	0.7 (0.5, 0.9)	83.7	64.1	57.7 (38.6, 82.3)	69.0
14–17	769	645 (403, 994)	36.8	0.8	0.7 (0.6, 0.9)	91.8	0.8	0.7 (0.5, 1.0)	85.9	65.7	59.3 (40.0, 84.3)	75.5

* μg retinol activity equivalent per day.

## Appendix A

**Table A1:** EARs or AIs for selected micronutrients for Chinese children *.

Items	Calcium	Iron	Zinc	Selenium	Sodium	Potassium	Vitamin A	Thiamine	Riboflavin	Vitamin C
	EAR (mg/Day)	EAR (mg/Day)	EAR (mg/Day)	EAR (mg/Day)	AI (mg/Day)	AI (mg/Day)	EAR (μg/Day) **	EAR (mg/Day)	EAR (mg/Day)	EAR (mg/Day)
Boys										
4–6	650	7	4.6	25	900	1200	260	0.6	0.6	40
7–10	800	10	5.9	35	1200	1500	360	0.8	0.8	55
11–13	1000	11	8.2	45	1400	1900	480	1.1	1.1	75
14–17	800	12	9.7	50	1600	2200	590	1.3	1.3	85
Girls										
4–6	650	7	4.6	25	900	1200	260	0.6	0.6	40
7–10	800	10	5.9	35	1200	1500	360	0.8	0.8	55
11–13	1000	14	7.6	45	1400	1900	450	1.0	0.9	75
14–17	800	14	6.9	50	1600	2200	450	1.1	1.0	85

* Chinese Dietary Reference Intakes (DRIs) 2013 [18]. ** μg retinol activity equivalent per day. EARs: Estimated Average Requirements; AI: Adequate Intake.
